# Human β‐defensin 3 increases the TLR9‐dependent response to bacterial DNA

**DOI:** 10.1002/eji.201646799

**Published:** 2017-02-14

**Authors:** Sarah L. McGlasson, Fiona Semple, Heather MacPherson, Mohini Gray, Donald J. Davidson, Julia R. Dorin

**Affiliations:** ^1^ MRC Human Genetics UnitIGMMUniversity of EdinburghEdinburghUK; ^2^ MRC Centre for Inflammation ResearchQMRIUniversity of EdinburghEdinburghUK

**Keywords:** Antimicrobial peptide, β‐Defensin, Dendritic cells, Plasmacytoid dendritic cells, Toll like receptor 9, Type I interferon

## Abstract

Human β‐defensin 3 (hBD3) is a cationic antimicrobial peptide with potent bactericidal activity in vitro. HBD3 is produced in response to pathogen challenge and can modulate immune responses. The amplified recognition of self‐DNA by human plasmacytoid dendritic cells has been previously reported, but we show here that hBD3 preferentially enhances the response to bacterial DNA in mouse Flt‐3 induced dendritic cells (FLDCs) and in human peripheral blood mononuclear cells. We show the effect is mediated through TLR9 and although hBD3 significantly increases the cellular uptake of both *E. coli* and self‐DNA in mouse FLDCs, only the response to bacterial DNA is enhanced. Liposome transfection also increases uptake of bacterial DNA and amplifies the TLR9‐dependent response. In contrast to hBD3, lipofection of self‐DNA enhances inflammatory signaling, but the response is predominantly TLR9‐independent. Together, these data show that hBD3 has a role in the innate immune‐mediated response to pathogen DNA, increasing inflammatory signaling and promoting activation of the adaptive immune system via antigen presenting cells including dendritic cells. Therefore, our data identify an additional immunomodulatory role for this copy‐number variable defensin, of relevance to host defence against infection and indicate a potential for the inclusion of HBD3 in pathogen DNA‐based vaccines.

## Introduction

Antimicrobial peptides (AMP) such as defensins and the cathelicidin LL‐37 were initially characterized as potent bactericidal agents in vitro. AMP are now also recognized as important immunomodulatory molecules, hence the term host defence peptide (HDP) is now widely used 1, 2. HDP are cationic peptides with amphipathic structures enabling them to rapidly enter cells 3, 4, 5. The expression of β‐defensins at mucosal surfaces is minimal until triggered by pathogen‐ or damage‐associated molecular patterns (PAMP/DAMP) or inflammation 6, 7. HDP have been shown to modulate the host responses to nucleic acids. Both LL‐37 and human β‐defensin 3 (hBD3) have been shown to amplify the type I interferon response to viral dsRNA, via the MAVS pathway in keratinocytes, and through MDA5/MAVS in macrophages 3, 8. LL‐37, complexed with endogenous self‐DNA, enhances activation of the cytoplasmic pattern recognition receptor stimulator of interferon genes in monocytes and TLR9 in human plasmacytoid dendritic cells (pDCs), resulting in a significant increase in production of the type I interferon, IFN‐α 9. Similarly, hBD3 has recently been shown to precipitate condensation of human self‐DNA, increasing its entry into pDCs and triggering IFN‐α production 10. Furthermore, hBD2 and hBD3 can enhance the uptake of self‐DNA and promote DNA‐induced IFN‐α expression in human pDCs 11. These immunomodulatory properties of HDP have implicated their involvement in the pathogenesis of autoimmune diseases including psoriasis, systemic lupus erythematous, and type I diabetes 10, 12, 13. Increased copy number of the hyper copy number variable human β‐defensin locus has been associated with psoriasis 14. However, bacterial infection is a relatively common occurrence compared to development of autoimmune disease. This highlights the importance of investigating the previously undescribed effect of hBD3 on the response to bacterial DNA. Production of hBD3 at the site of infection results in bacterial cell wall disruption and osmotic lysis, leading to release of cytoplasmic contents, including DNA 15, 16. The increased response of antigen presenting cells to pathogen DNA in the presence of hBD3 would promote the functional activation of other immune cells involved in host defence. Therefore, we sought to determine the cellular response of mouse and human antigen presenting cells to bacterial DNA in the presence of hBD3 as would be encountered in an infection setting.

We show here that hBD3 significantly amplified the response to bacterial DNA in both mouse and human immune cells in a TLR9‐dependent manner. This demonstrates a further important aspect of protection from infection afforded by defensins and suggests that this property of hBD3 could be utilized to increase efficacy of DNA‐based vaccines.

## Results and discussion

### hBD3 enhances the immune response to bacterial DNA in murine FLDCs and human PBMCs

The effect of hBD3 on the immune response to bacterial DNA, such as would be released by AMP‐mediated killing during infection was investigated in murine Flt‐3 ligand‐induced bone marrow‐derived dendritic cell cultures (FLDCs), comprising a mixed population of conventional DCs (cDCs) and plasmacytoid DCs (pDCs). FLDCs have been widely used to study the innate immune response to nucleic acids 11, 17, 18. cDCs and pDCs are identifiable by expression of CD11c and B220 and activation of the populations can be independently assessed (Supporting Information Fig. 1). pDCs (CD11c^+^ B220^high^), constitute approximately 10–20% of FLDCs and are specialized to rapidly produce large amounts of IFN‐α in response to particular immune stimuli, due to increased expression and rapid activation of the transcription factor IFN regulatory factor 7, by a pDC‐specific MyD88‐dependent pathway 19, 20.

**Figure 1:**
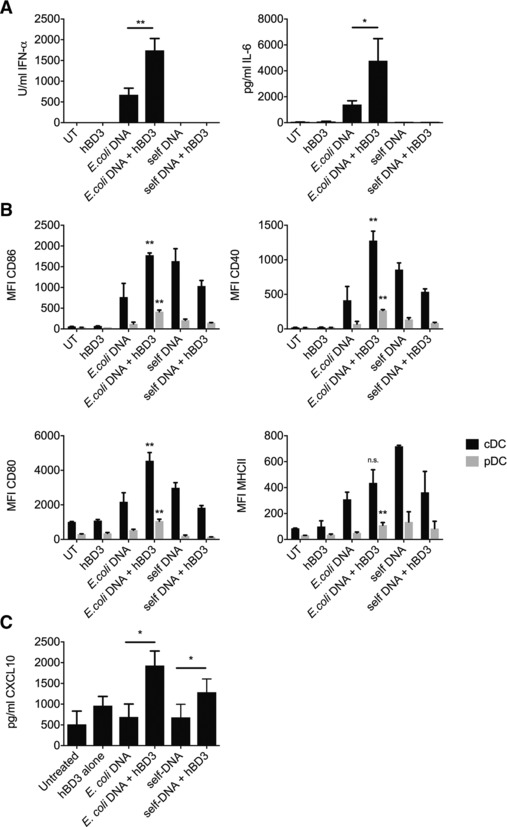
hBD3 stimulates an increased inflammatory response to bacterial DNA in mouse FLDCs and human PBMCs. Mouse FLDCs were incubated with *E. coli*‐ or self‐DNA with or without hBD3. Cells and supernatants were collected. IFN‐α and IL‐6 secretion were assayed by ELISA (A) and costimulatory marker expression was assayed in cDCs (CD11c^+^ B220^−^) and pDCs (CD11c^+^ B220^high^) by flow cytometry (B). Data are shown as mean ± SEM and are pooled from three or more independent experiments performed in triplicate. *p < 0.05, **p < 0.01 unpaired *t*‐test. Human PBMCs were extracted from peripheral blood by Ficoll gradient centrifugation. PBMCs were immediately incubated with *E. coli*‐ or self‐DNA with or without hBD3. Supernatants were harvested after 24 h and CXCL10 measured by ELISA (C). Data are shown as mean ± SEM and are pooled from five independent experiments with one donor sample per experiment. *p < 0.05 paired *t*‐test.

As expected, treatment of murine FLDCs with 1 μg/mL *E. coli*‐DNA resulted in upregulation of costimulatory molecules and production of IFN‐α and IL‐6 (Fig. 1A and B). We confirmed that the response to *E. coli*‐DNA was not caused by another bacterial contaminant in the DNA preparation (Supporting Information Fig. 2).

HBD3 alone had no effect on cytokine expression, cell surface marker expression or viability of FLDCs at the concentration used throughout this study (Supporting Information Fig. 3). However, 5 μg/mL hBD3 (1 μM) in combination with *E. coli*‐DNA resulted in a significant increase in the production of IFN‐α and IL‐6 and the expression of costimulatory molecules by murine FLDCs compared with bacterial DNA alone (Fig. 1A and B and Supporting Information Fig. 4). We additionally show that hBD3 combined with bacterial DNA, significantly increased the production of CXCL10 (a cytokine induced by type I interferon and reflects activation of pDCs 21) by human ex vivo PBMCs (Fig. 1C). It is therefore likely that the bacterial DNA‐induced cytokine release we observe in human (as in mouse) is due to TLR9 activation of pDCs in the total PBMC population. This has previously been shown with CpG in human pDCs isolated from peripheral blood 22. Human cDCs do not express TLR9 22 but other immune cells in human PBMCs do express TLR9 (NK cells; B cells; CD4+ and CD8+ T cells and predominantly CD14 monocytes 23) and these may also be activated and contribute to the increased cytokine release we observe with bacterial DNA in combination with hBD3.

### hBD3 enhances the immune response to self‐DNA only in human PBMCs

It has been previously reported that self‐DNA alone does not induce any response in human pDCs. However liposome transfection 24 or complexing to HDP (including hBD3) increases the IFN‐α response to self‐DNA in human pDCs 10, 11. Schmidt et al. showed that various cationic molecules enable endosomal localization of self‐DNA, thereby increasing access to endosomal TLR9 in human pDCs 25. However only molecules of a certain size enabled optimal inter‐DNA spacing allowing multiple TLR9 interactions and activation that correlated with an increased interferon response and hBD3 allowed optimal arrangement of DNA ligand and human TLR9 25. In agreement with these publications, we see an increase in CXCL10 in human pDCs treated with self‐DNA and hBD3 compared to self‐DNA alone. In contrast, in mouse FLDCs, self‐DNA did not stimulate an immune response and hBD3 did not influence this (Fig. 1A–C). Defb14 is the clear orthologue of DEFB103 (hBD3) despite being only 64% identical and we have previously demonstrated that its bactericidal and chemoattractant abilities are fundamentally similar 26. In addition, Barabas et al. have shown that mouse bone marrow derived macrophages exposed to Defb14 reveal an exacerbated response to various TLR ligands, including CpG that is known to activate TLR9. This implies that the increased TLR9 response to bacterial DNA by these defensin peptides is conserved between human and mouse 27. However, the species difference in the influence of hBD3 on the response of pDCs to self‐DNA may reflect the differences between human and mouse in the ability of TLR9 to recognize mammalian DNA 28.

### The increased immune response of FLDCs to bacterial DNA in the presence of hBD3 is mediated by TLR9

**Figure 2:**
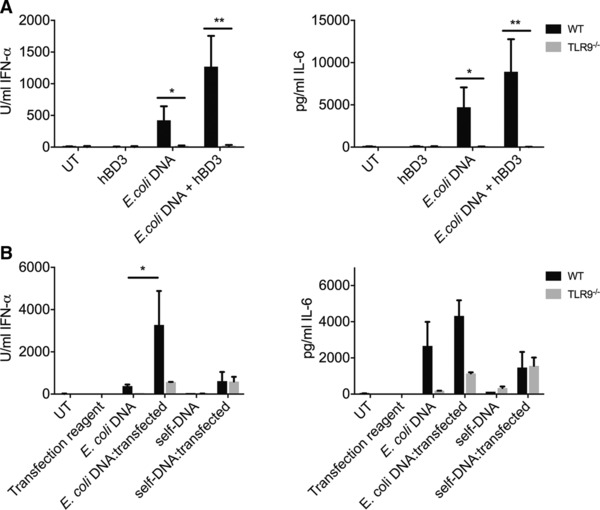
The effect of hBD3 on the response to *E. coli*‐DNA in mouse FLDCs is TLR9 dependent. Mouse FLDCs from WT or TLR9 deficient (TLR9^−/−^) mice were incubated with *E. coli*‐DNA with or without hBD3. Supernatants were collected and cytokine secretion was assayed by ELISA (A). Mouse FLDCs from WT or TLR9 deficient (TLR9^−/−^) mice were incubated or transfected with *E. coli*‐ or self‐DNA. Supernatants were collected and cytokine secretion was assayed by ELISA (B). The response to TLR4 ligand LPS was intact in both WT and TLR9 –/–FLDC (and reduced in the presence of hBD3 as expected [31](Supporting Information Fig.6), Data are shown as mean ± SEM and are pooled from three or more independent experiments performed in triplicate. *p < 0.05, **p < 0.01 unpaired *t*‐test.

We used TLR9^−/−^ FLDCs to test whether the hBD3‐mediated amplification of the immune response of FLDCs to bacterial DNA was due to increased activation of TLR9, or due to activation of an alternative cytoplasmic pattern recognition receptor, as we have previously shown with hBD3 and poly(I:C)) 3. Endosomal TLR9 is activated by bacterial DNA due to the presence of unmethylated CpG residues, and can also sense the phosphodiester DNA backbone of unmodified single‐stranded DNA in a CpG‐independent manner 29, 30. FLDCs treated with in vitro methylated *E. coli*‐DNA showed a significant reduction in the inflammatory response compared to exposure to untreated *E. coli*‐DNA (Supporting Information Fig. 5) consistent with TLR9 involvement. In addition, the cytokine response to bacterial DNA with or without hBD3 was ablated in TLR9^−/−^ FLDCs (Fig. 2A). These results confirm that the increased response of FLDCs to bacterial DNA when in the presence of hBD3 is mediated through TLR9.

We showed that self‐DNA could only stimulate an immune response in FLDCs when transfected (Fig. 2B). This response to transfected self‐DNA was shown to be almost entirely TLR9‐independent implicating an alternative receptor in the response of FLDCs to transfected self‐DNA (Fig. 2B). This is in contrast, to the response to transfected bacterial DNA, which was almost entirely TLR9‐dependent (Fig. 2B). These results firstly confirm that lipofection transports DNA via the endosome since the response to bacterial DNA is TLR9‐dependent. Secondly, this demonstrates that in murine FLDCs, self‐DNA complexed with lipofectamine is not recognized by TLR9, and likely activates a cytoplasmic DNA receptor upon escape from the endosomal pathway.

### hBD3 increases uptake of labeled *E. coli*‐DNA and self‐DNA into FLDCs

We next sought to understand the mechanistic basis for the effect of hBD3 on the response to bacterial DNA. An electrophoretic mobility shift assay demonstrated that hBD3 prevented migration of DNA into an agarose gel in concentration‐dependent manner (Supporting Information Fig. 7). This suggests that, in vitro, hBD3 causes DNA to aggregate into a complex large enough to prevent migration into a gel.

**Figure 3:**
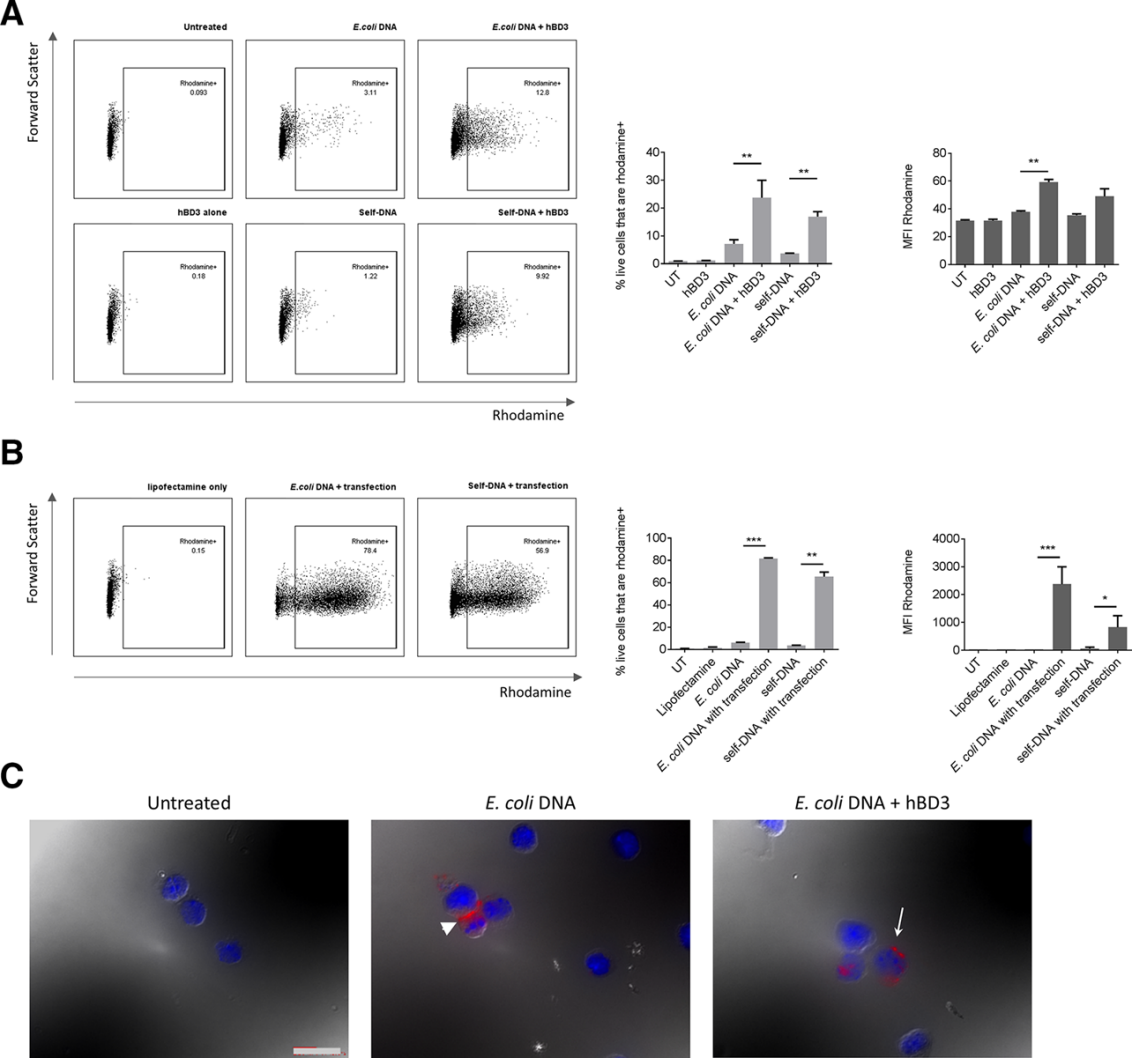
hBD3 causes increased uptake of labeled *E. coli*‐ and self‐DNA into mouse FLDCs and aggregation of DNA. Mouse FLDCs were incubated or transfected with rhodamine‐labeled *E. coli*‐DNA or rhodamine‐labeled self‐DNA with or without hBD3, or lipofection. Cell uptake was assayed by flow cytometry. Representative flow plots show gating of Rhodamine^+^ cells. DNA uptake was quantified by measuring proportion of rhodamine+ cells and MFI following incubation of cells with labeled DNA and hBD3 (A) or lipofectamine. (B) Data are shown as mean ± SD and are pooled from two independent experiments. *p < 0.05, **p < 0.01, ***p < 0.005 unpaired *t*‐test. Uptake of labeled *E. coli*‐DNA was visualized by fluorescence and differential interference contrast microscopy. White arrow indicates intracellular labeled *E. coli*‐DNA, in contrast to white arrow head indicating extracellular DNA (C). Scale bar = 400 μm. Images are from a single experiment representative of two independent experiments.

We used fluorescently labeled *E. coli‐*DNA to further investigate the effect hBD3 has on the cellular uptake of bacterial DNA. Flow cytometry demonstrated that hBD3 increased uptake of fluorescently labeled bacterial DNA by FLDCs, (Fig. 3A (MFI)). Interestingly, the proportion of cells taking up fluorescently labeled self‐DNA was also significantly increased in the presence of hBD3 (Fig. 3A (%Rhodamine^+^)) suggesting that, despite hBD3 increasing uptake of bacterial and self‐DNA, self‐DNA is intrinsically nonimmunogenic in mouse FLDCs and does not activate any immune receptors in these cells.

We additionally observed by microscopy that hBD3 treatment appeared to result in DNA aggregation in FLDCs compared with a more diffuse pattern in the absence of hBD3 (Fig. 3C, white arrows). This corresponds with the results from the gel shift assay. These data indicate that hBD3 increases the response to *E. coli*‐DNA by increasing cellular uptake, leading to increased activation of TLR9 and a consequent increase in costimulatory marker expression and cytokine production.

### Concluding remarks

In summary, we show that in both mouse and human immune cells the antimicrobial host defence peptide hBD3 amplifies the response to bacterial DNA, eliciting increased expression of costimulatory markers and inflammatory cytokines, including type I interferons. The hBD3 gene, *DEFB103*, is present in a highly copy number variable locus in humans and copy number of this locus correlates with systemic peptide expression 32. Our work here suggests that copy number may influence the individual's response to infection. Additionally, this work highlights future potential for hBD3 as a TLR9 agonist for novel DNA‐based vaccine therapeutics, acting by sensitizing the immune response, particularly to pathogen components.

## Materials and methods

### Antibodies and reagents

*E. coli* genomic DNA (InvivoGen) was used at 1 μg/mL. Mouse genomic DNA was used at 1 μg/mL. hBD3 (Peptide Institute) was used at 5 μg/mL. Lipofectamine LTX (ThermoFisher) was used as per manufacturer's instructions.

The following antibodies were used for flow cytometry: CD11c‐APC‐eFluor780 (eBioscience), B220‐eFluor450 (eBioscience), CD80‐APC (eBioscience), CD86‐AlexaFluor488 (Biolegend), CD40‐PE (BD Bioscience), MHCII‐PerCP‐Cy5.5 (Biolegend).

Murine IL‐6 and human IP‐10 were quantified using Duoset kits (R&D). Murine IFN‐α was quantified using the following: Rat anti‐mouse IFN‐α MAb (capture antibody), Rabbit anti‐mouse IFN‐α PAb (detection antibody), Recombinant mouse IFN‐α A (standard) (all PBL Interferon Source), HRP‐conjugated donkey anti‐rabbit (secondary antibody, Jackson Immunoresearch).

DNA was fluorescently labelled using the Label‐IT nucleic acid labeling kit (MirusBio).

Cell viability was measured by quantifying lactate dehydrogenase (LDH) activity in cell supernatants using an LDH‐cytotoxicity assay kit (Biovision).

The effect of hBD3 on migration of DNA into an agarose gel was determined by incubating hBD3 and DNA and running the entire reaction into a low percentage (0.5%) agarose gel containing ethidium bromide. The gel was poststained with Coomassie Blue.

### FLDC isolation and culture

Bone marrow was extracted from the femurs of 8–12 week old wild‐type C57Bl6/J mice or TLR9^−/−^ mice (with littermates as controls) 29 and cultured with Flt‐3L (200 ng/mL, Peprotech) for 8 days. Animal studies were covered by a Project License, granted by the UK Home Office under the Animal Scientific Procedures Act 1986, and locally approved by the University of Edinburgh Ethical Review Committee.

### PBMC isolation

PBMCs were isolated from peripheral blood of adult donors using Ficoll gradient and plated for immediate treatment. Human venous blood was collected with written patient consent from healthy volunteers according to Lothian Research Ethics Committee approvals under AMREC Reference number 15‐HV‐013.

### Cell stimulation

Cells were harvested and plated at 2.5 × 10^6^ cells/mL, and incubated with ligands for 18–24 h. Supernatants were collected for analysis by ELISA. Cells were collected and stained for analysis by flow cytometry. Data were acquired using FACS Aria II (BD Biosciences) and analyzed using FlowJo v_9/10. For fluorescent microscopy, cells were treated for 4 h then harvested and attached to poly‐lysine slides. Cells were fixed in 4% PFA and mounted using Vectashield with DAPI (VECTOR laboratories).

### Statistical tests

Unpaired Student's *t*‐tests were performed unless otherwise stated, calculated using GraphPad Prism Software version 6/7 (La Jolla, USA). Significance levels are denoted in figures as: *p ≤ 0.05, **p ≤ 0.01, ***p ≤ 0.001, n.s. not significant.

## Conflict of interest

The authors declare no financial or commercial conflict of interest.

**Abbreviations**
AMP: antimicrobial peptide
cDC: conventional dendritic cells
HDP: host defence peptide
hBD3: human β‐defensin 3
FLDC: FLT‐3 ligand induced dendritic cells
pDC: plasmacytoid dendritic cell

## Supporting information


**Supplementary Information Figure S1**. Representation of flow cytometric analysis of FLDC. Single cells were gated from debris and clumps based on forward and side scatter. CD11c+ cells were gated into B220+ pDCs and B220‐ cDCs. The expression of surface costimulatory markers was analysed on each population and quantified as Median Fluorescence Intensity (MFI) across the entire population (CD86 is shown as an example here).
**Supplementary Information Figure S2**. The immune response to E. coli‐DNA is not due to a preparation contaminant. The E. coli DNA solution was treated with DNase I and degradation was confirmed by gel electrophoresis (A). FLDC were incubated with DNase I treated or untreated E. coli‐DNA for 18 hours and supernatants were collected for analysis by ELISA (B). Data shown is the mean of 2 independent experiments performed in triplicate ± standard error.
**Supplementary Information Figure S3**. hBD3 alone, at concentration used in this study (5 ìg/ml) has no effect on the relative proportion of FLDC populations, the expression of costimulatory markers or on the viability of the cells. FLDC were incubated with 5 ìg/ml hBD3 for 18 hours. Cells were collected for analysis by flow cytometry (A, B) and supernatants were collected for analysis of viability by lactate dehydrogenase assay (C). Data shown is the mean of 3 independent experiments performed in triplicate ± SEM. NB: ‘P1’ is the first population gated on the basis of forward and side scatter and removes cell clumps and debris from subsequent analysis.
**Supplementary Information Figure S4**. 5 ìg/ml hBD3 in combination with 1 ìg/ml E. coli DNA showed the largest increase in response compared with E. coli DNA alone. FLDC were incubated with 1 ìg/ml E. coli DNA and increasing concentration hBD3 for 18 hours. Supernatants were collected for ELISA assays. Data shown is one independent experiment performed in triplicate ±SD.
**Supplementary Information Figure S5**. Methylation suppresses the response to pathogen DNA but hBD3 still exacerbates the response. E.coli‐DNA was incubated withM.SssI methylase. A dot blot was performed to detect methlyated CpG motifs in CpG1585 (unmethylated), mouse genomic DNA, Interferon Stimulatory DNA and treated or untreated E. coli‐DNA. Equivalent amounts of each oligonucleotide was applied to a nitrocellulose membrane and probed with anti‐5‐methylcytosine (5‐mC) antibody (Abcam) (A). FLDC were incubated with E. coli‐DNA which had been treated with M.SssI with or without hBD3 and supernatants were collected for analysis by ELISA (B). Data shown is the mean of 3 independent experiments performed in triplicate ± standard error. *p < 0.05, **p < 0.01.
**Supplementary Information Figure S6**. TLR9‐/‐ FLDCs respond as expected to the TLR4 ligand, LPS. Mouse FLDC from WT or TLR9 deficient (TLR9‐/‐) mice were incubated with LPS (1 ƒÝg/ml) with or without hBD3 (5 ƒÝg/ml) for 18 hours. Supernatants were collected and IL‐6 secretion was assayed by ELISA. Data is the mean of 2 independent experiments performed in triplicate ± SD.
**Supplementary Information Figure S7**. hBD3 prevents migration of DNA into an agarose gel in a concentration dependent manner. E. coli‐DNA or self‐DNA was incubated with hBD3 at varying molar ratios in serum‐free media (Optimem) or in 8% serum to recapitulate culture conditions. The total reaction volume was then analysed by gel electrophoresis (A). The gel was post‐stained with Coomassie blue protein stain to identify whether hBD3 was associated with trapped DNA aggregates (B).

Peer review correspondence
