# Supplementary material for: Human β‐defensin 3 increases the TLR9‐dependent response to bacterial DNA
Source: Eur J Immunol. 2017 Feb 14;47(4):658–64. doi: 10.1002/eji.201646799 (PMC5412915; doi:10.1002/eji.201646799)

European Journal of Immunology

Supporting Information
for

DOI 10.1002/eji.201646799

Sarah L. McGlasson, Fiona Semple, Heather MacPherson, Mohini Gray, Donald J. Davidson and Julia R. Dorin

Human β-defensin 3 increases the TLR9-dependent response to bacterial DNA

© 2017 The Authors. *European Journal of Immunology* published by WILEY-VCH Verlag GmbH & Co. KGaA, Weinheim.

Supplementary Figure 1: Representation of flow cytometric analysis of FLDC.

Single cells were gated from debris and clumps based on forward and side scatter. CD11c^{+} cells were gated into B220^{+} pDCs and B220^{-} cDCs. The expression of surface costimulatory markers was analysed on each population and quantified as Median Fluorescence Intensity (MFI) across the entire population (CD86 is shown as an example here).

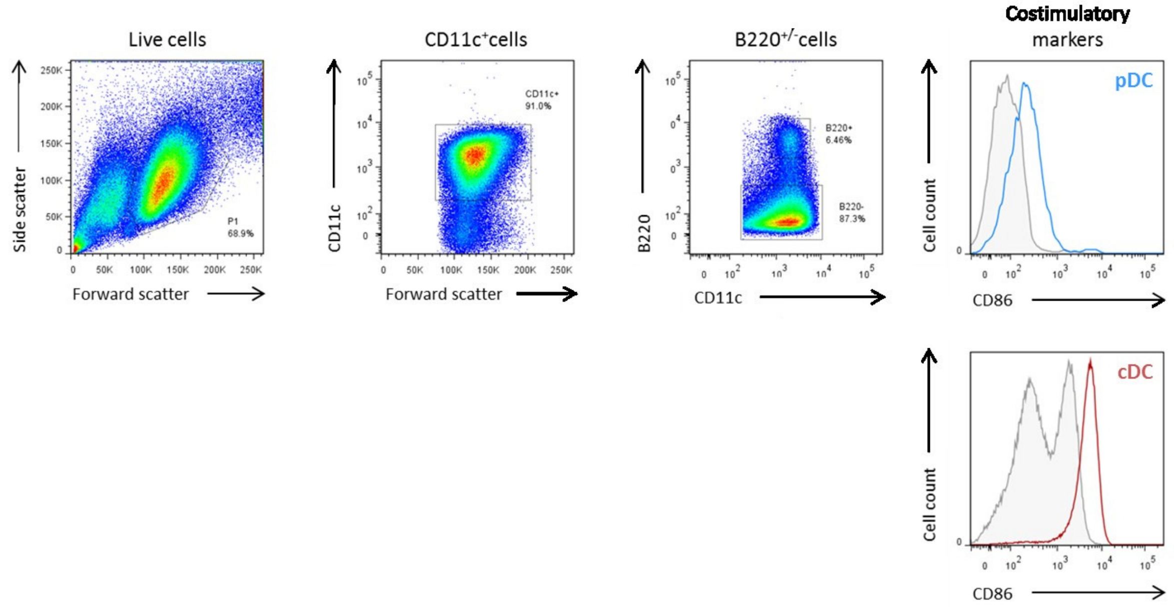

Supplementary Fig 2: The immune response to E. coli-DNA is not due to a preparation contaminant. *The E. coli DNA solution was treated with DNase I and degradation was confirmed by gel electrophoresis (A). FLDC were incubated with DNase I treated or untreated E. coli-DNA for 18 hours and supernatants were collected for analysis by ELISA (B). Data shown is the mean of 2 independent experiments performed in triplicate ± standard error.*

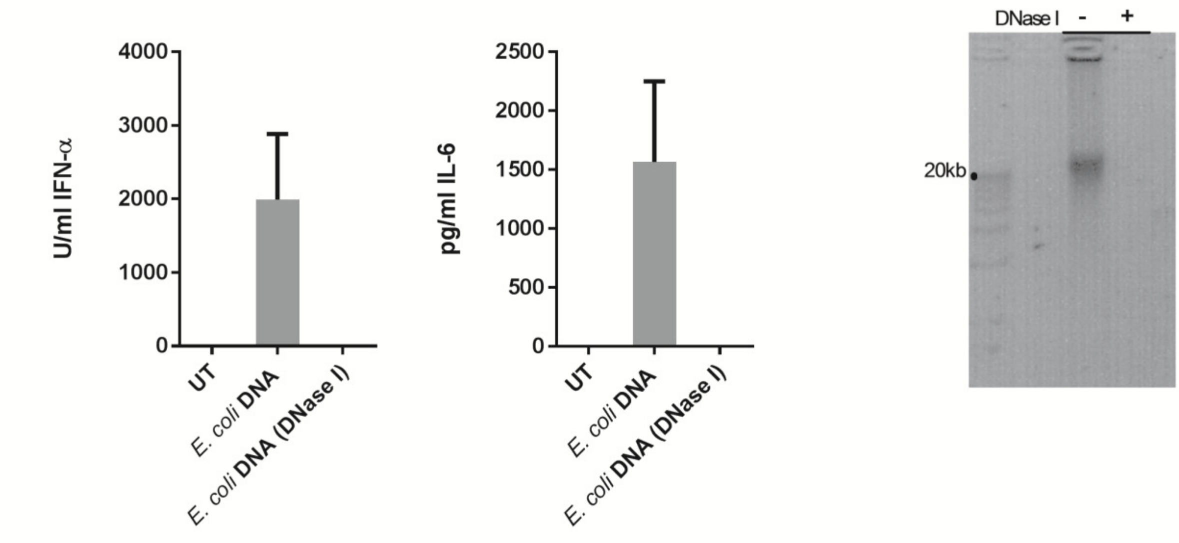

Supplementary figure 3: hBD3 alone, at concentration used in this study (5 µg/ml) has no effect on the relative proportion of FLDC populations, the expression of costimulatory markers or on the viability of the cells. *FLDC were incubated with 5 µg/ml hBD3 for 18 hours. Cells were collected for analysis by flow cytometry (A, B) and supernatants were collected for analysis of viability by lactate dehydrogenase assay (C). Data shown is the mean of 3 independent experiments performed in triplicate ± SEM. NB: 'P1' is the first population gated on the basis of forward and side scatter and removes cell clumps and debris from subsequent analysis.*

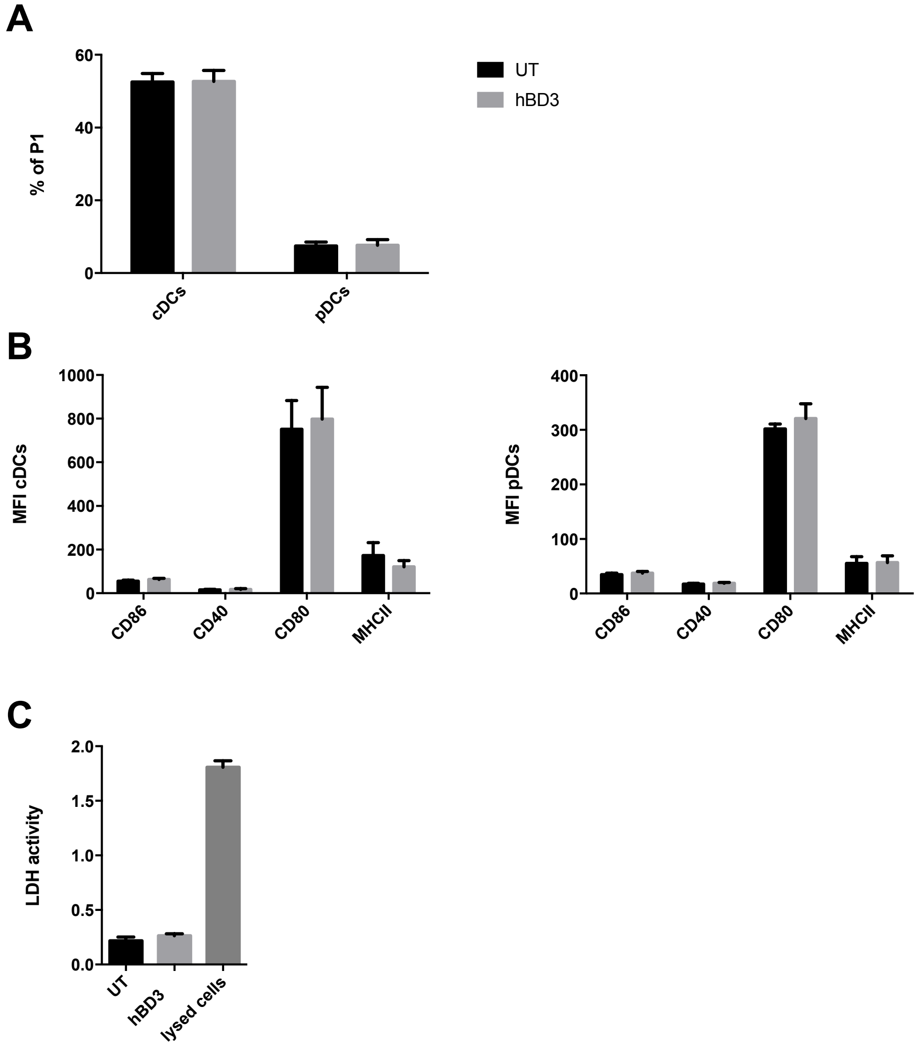

Supplementary Figure 4: 5 µg/ml hBD3 in combination with 1 µg/ml E. coli DNA showed the largest increase in response compared with E. coli DNA alone.

FLDC were incubated with 1 µg/ml E. coli DNA and increasing concentration hBD3 for 18 hours. Supernatants were collected for ELISA assays. Data shown is one independent experiment performed in triplicate ±SD.

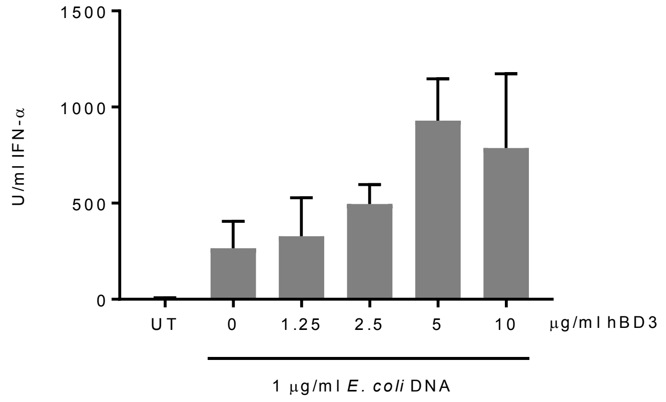

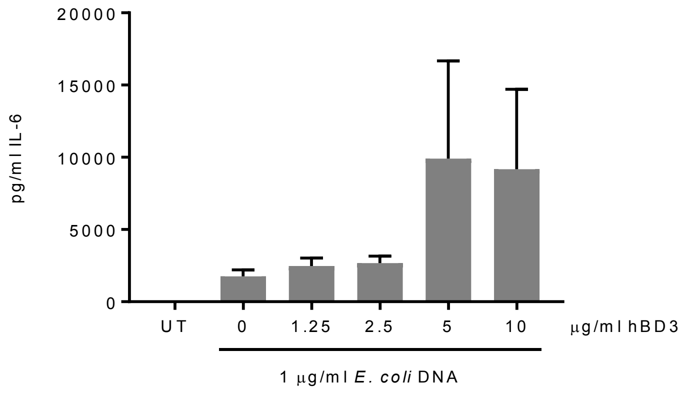

Supplementary Figure 5: Methylation suppresses the response to pathogen DNA but hBD3 still exacerbates the response. *E.coli-DNA was incubated with M.SssI methylase. A dot blot was performed to detect methlyated CpG motifs in CpG1585 (unmethylated), mouse genomic DNA, Interferon Stimulatory DNA and treated or untreated E. coli-DNA. Equivalent amounts of each oligonucleotide was applied to a nitrocellulose membrane and probed with anti-5-methylcytosine (5-mC) antibody (Abcam) (A). FLDC were incubated with E. coli-DNA which had been treated with M.SssI with or without hBD3 and supernatants were collected for analysis by ELISA (B). Data shown is the mean of 3 independent experiments performed in triplicate ± standard error. *p<0.05, **p<0.01*

A

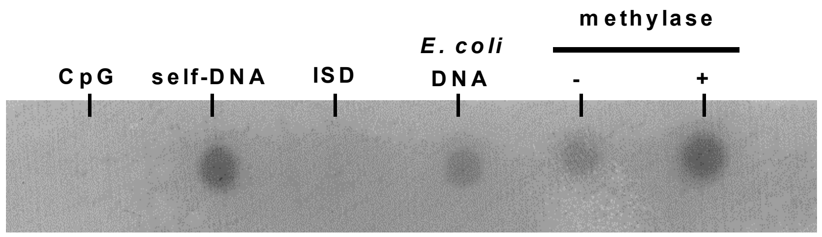

B

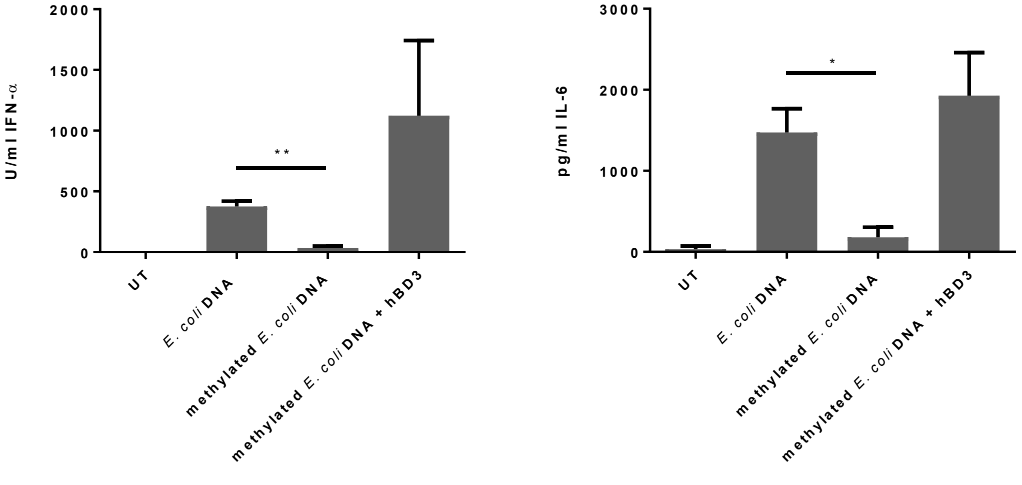

Supplementary Figure 6: TLR9$^{-/-}$ FLDCs respond as expected to the TLR4 ligand, LPS. *Mouse FLDC from WT or TLR9 deficient (TLR9$^{-/-}$) mice were incubated with LPS (1 μg/ml) with or without hBD3 (5 μg/ml) for 18 hours. Supernatants were collected and IL-6 secretion was assayed by ELISA. Data is the mean of 2 independent experiments performed in triplicate ± SD*

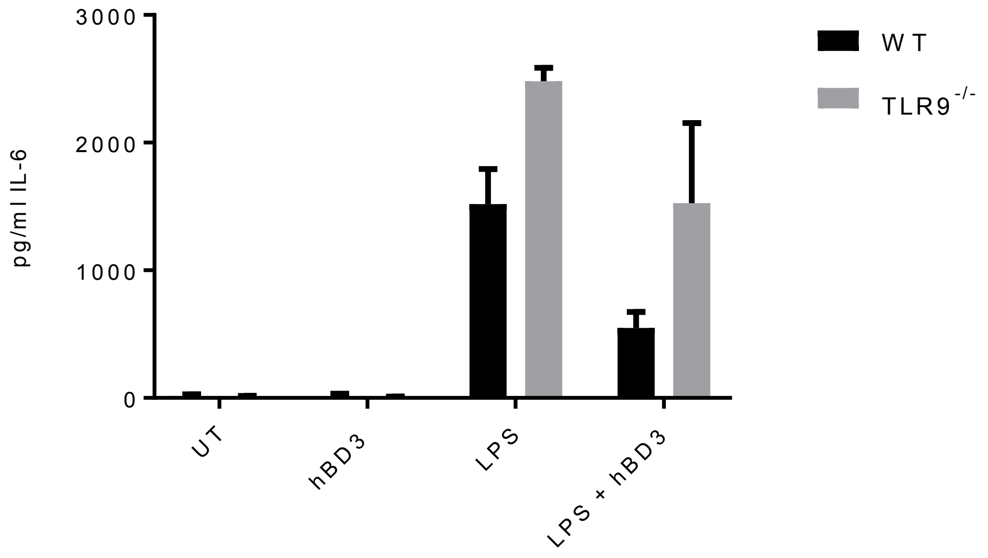

Supplementary Figure 7: hBD3 prevents migration of DNA into an agarose gel in a concentration dependent manner. *E. coli-DNA or self-DNA was incubated with hBD3 at varying molar ratios in serum-free media (Optimem) or in 8% serum to recapitulate culture conditions. The total reaction volume was then analysed by gel electrophoresis (A). The gel was post-stained with Coomassie blue protein stain to identify whether hBD3 was associated with trapped DNA aggregates (B).*

A

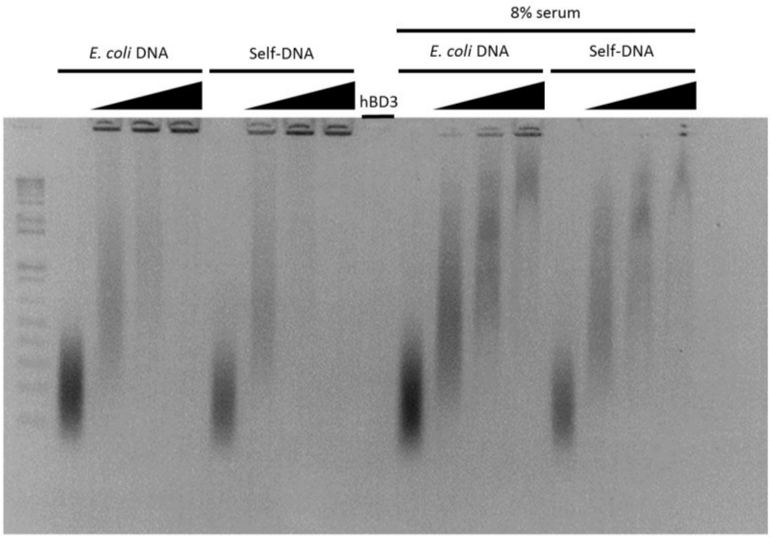

B

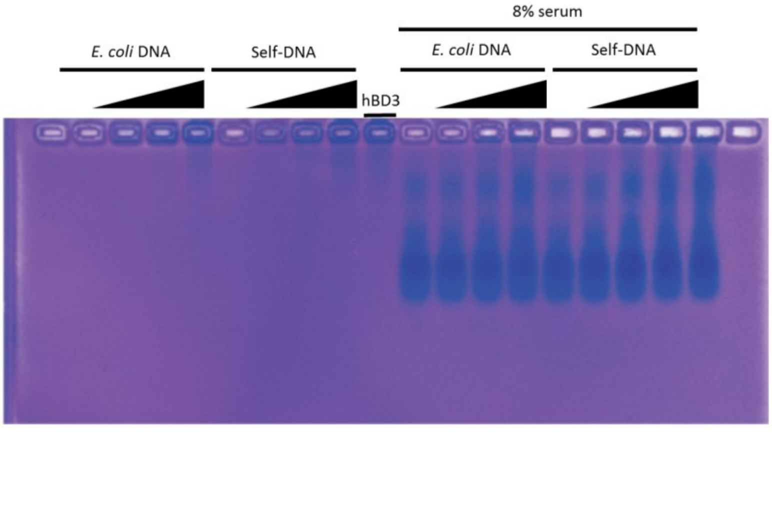

Supplement: Supplementary file 1 — Supplementary Information Figure S1. Representation of flow cytometric analysis of FLDC. Single cells were gated from debris and clumps based on forward and side scatter. CD11c+ cells were gated into B220+ pDCs and B220‐ cDCs. The expression of surface costimulatory markers was analysed on each population and quantified as Median Fluorescence Intensity (MFI) across the entire population (CD86 is shown as an example here). Supplementary Information Figure S2. The immune response to E. coli‐DNA is not due to a preparation contaminant. The E. coli DNA solution was treated with DNase I and degradation was confirmed by gel electrophoresis (A). FLDC were incubated with DNase I treated or untreated E. coli‐DNA for 18 hours and supernatants were collected for analysis by ELISA (B). Data shown is the mean of 2 independent experiments performed in triplicate ± standard error. Supplementary Information Figure S3. hBD3 alone, at concentration used in this study (5 ìg/ml) has no effect on the relative proportion of FLDC populations, the expression of costimulatory markers or on the viability of the cells. FLDC were incubated with 5 ìg/ml hBD3 for 18 hours. Cells were collected for analysis by flow cytometry (A, B) and supernatants were collected for analysis of viability by lactate dehydrogenase assay (C). Data shown is the mean of 3 independent experiments performed in triplicate ± SEM. NB: ‘P1’ is the first population gated on the basis of forward and side scatter and removes cell clumps and debris from subsequent analysis. Supplementary Information Figure S4. 5 ìg/ml hBD3 in combination with 1 ìg/ml E. coli DNA showed the largest increase in response compared with E. coli DNA alone. FLDC were incubated with 1 ìg/ml E. coli DNA and increasing concentration hBD3 for 18 hours. Supernatants were collected for ELISA assays. Data shown is one independent experiment performed in triplicate ±SD. Supplementary Information Figure S5. Methylation suppresses the response to pathogen DNA [file EJI-47-658-s001.pdf]
