# Supplementary material for: Human β‐defensin 3 increases the TLR9‐dependent response to bacterial DNA
Source: Eur J Immunol. 2017 Feb 14;47(4):658–64. doi: 10.1002/eji.201646799 (PMC5412915; doi:10.1002/eji.201646799)
Supplement: Supplementary file 2 — Peer review correspondence [file EJI-47-658-s002.pdf]

Human β-defensin 3 increases the TLR9-dependent response to bacterial DNA

Sarah L. McGlasson, Fiona Semple, Heather MacPherson, Mohini Gray, Donald J. Davidson, and Julia R. Dorin

Correspondence: Dr. Julia Dorin, MRC Centre for Inflammation Research, University of Edinburgh, QMRI, Edinburgh, UK

Review Timeline:	Submission date:	08-Nov-2016
	First Editorial decision:	15-Dec-2016
	Revision received:	22-Dec-2016
	Second Editorial decision:	10-Jan-2017
	Accepted:	17-Jan-2017

Handling Executive Committee member: Prof. Kenneth Murphy

Please note that the correspondence below does not include the standard editorial instructions regarding preparation and submission of revised manuscripts, only the scientific revisions requested and addressed.

For Peer Review

First Editorial Decision

15-Dec-2016

Dear Dr. Dorin,

Manuscript ID eji.201646799 entitled "Human Î²-defensin 3 increases the TLR9-dependent response of plasmacytoid dendritic cells to bacterial DNA." which you submitted to the European Journal of Immunology has been reviewed. The comments of the referees are included at the bottom of this letter.

A revised version of your manuscript that takes into account the comments of the referees will be reconsidered for publication. Should you disagree with any of the referees concerns, you should address this in your point-by-point response and provide solid scientific reasons for why you will not make the requested changes.

Executive Editor: Reviewer 2 noted a major problem of sample cell heterogeneity. The editor agrees with this major technical problem, and would also raise to the authors a requirement for the inclusion of positive controls in Figure 2 for the ability of TLR9 KO samples to react to other TLR ligands, as a control at least for cell viability. It is a basic specificity control that is required.

Also, for the issue of sample heterogeneity, it would be highly beneficial to offer some additional data on the issue of in which cells this mechanisms is operative. Also, note that FACS profiles are not quantitative unless gates and percentages are included. The data in Fig. 3A is qualitative and not appropriate for publication.

You should also pay close attention to the editorial comments included below. **In particular, please edit your figure legends to follow Journal standards as outlined in the editorial comments. Failure to do this will result in delays in the re-review process.**

Please note that submitting a revision of your manuscript does not guarantee eventual acceptance, and that your revision will be re-reviewed by the referee(s) before a decision is rendered.

If the revision of the paper is expected to take more than three months, please inform the editorial office. Revisions taking longer than six months may be assessed by new referee(s) to ensure the relevance and timeliness of the data.

Once again, thank you for submitting your manuscript to European Journal of Immunology and we look forward to receiving your revision.

Yours sincerely,
Nadja Bakocevic

On behalf of
Prof. Kenneth Murphy

Dr. Nadja Bakocevic
Editorial Office
European Journal of Immunology
e-mail: ejied@wiley.com
www.eji-journal.eu

For Peer Review

****

Reviewer: 1

Comments to the Author

The authors have described ability of human hBD3 in immune responses to DNA via TLR9. The results in this manuscript expand upon the functional role of beta defensins in innate immunity.

Major comment:

The authors have demonstrated that human beta defenisn hBD3 enhances the immune response to self-DNA only in human PBMC, and not in murine cells. The murine ortholog beta defensin-14 has approx 65% sequence identity with the hDB3. So the question is will the murine BD-14 exhibit similar effects in murine cells, as seen with hBD3 in human cells? If so, this would support the functional role of these defensins in innate immune response to mammalian DNA. Either the authors can present some data with the murine BD-14 on murine cells, or if there is previous literature published discuss the effects of murine BD-14 on murine cells in the context of the data presented with hBD3 in human cells. This I believe would significantly strengthen the thesis of this manuscript.

Minor comments:

1. On Page 1, in the introduction, references to support the sentence HDP are cationic peptides with amphipathic structures enabling them to rapidly enter cells• need to include other HDPs and not just hDB3.

2. Please provide a rationale for the concentrations used for the stimulants, i.e. 1 ug/ml for genomic DNA and 5 ug/ml for hBD3, in this manuscript.

3. In Supplementary Figure 3: replace at the concentration used throughout this study with the concentration in parenthesis (5ug/ml).

Reviewer: 2

Comments to the Author

The authors have analyzed the role of the antimicrobial peptide HBD3 in promoting bacterial DNA mediated immune responses by mouse Flt 3 ligand induced DCs and human PBMCs. They show that

For Peer Review

HBD3 and bacterial DNA complexes activate these cell types in a TLR 9 dependent manner.

Major Comment : The authors have shown effect of bacterial DNA (containing DNA sequences similar to CpG DNA) in combination with HBD 3 to activate conventional DCs (cDCs) and plasmacytoid DCs(pDCs). However data with human cells are with PBMCs rather than purified cDCs. To date human cDCs have not been shown to express TLR-9. Thus it is likely that HBD 3 and DNA complexes are stimulating other TLR 9 expressing or CpG DNA detecting cells (B cells, NK cells pDCs and minor monocytic populations). The authors fail to mention or analyze these cell types. The results from human cells are cytokine responses from total PBMCs. Unlike the murine DCs the human cells have not been phenotypically characterized in the above article. Therefore it is difficult to deduce from the current experiments whether HBD3/bacterial complexes mediate activation of human cDCS /B cells or pDCs.

Minor comments.

1. Cell surface marker and gating strategies and for pDC and cDCs are inadequate. Conventional DCs are generally B220 negative rather than B220 low.
2. Supplementary figure 1. Expression of costimulatory molecules is not clear as to which population is being analyzed (cDCs or pDCs)
3. HBD 3 mediated complex/aggregate formation of CpG DNA (similar to bacterial DNA) has already been documented, hence the data is a confirmation of previously published work.

For Peer Review

<u>First Revision – authors' response</u>

<u>22-Dec-2016</u>

Dear Dr. Nadja Bakocevic/Prof. ken murphy,

Thank you for sending the reviewers comments on our paper and we are very glad that a revised version of our manuscript that takes into account the comments of the referees will be reconsidered for publication. Please find below our response to the reviewer's comments:

1. As noted by the Executive Editor, Reviewer 2 noted a major problem of sample cell heterogeneity. They specifically said that

"*data with human cells are with PBMCs rather than purified cDCs. To date human cDCs have not been shown to express TLR-9. Thus it is likely that HBD 3 and DNA complexes are*

stimulating other TLR 9 expressing or CpG DNA detecting cells (B cells, NK cells pDCs and minor monocytic populations). The authors fail to mention or analyze these cell types. The results from human cells are cytokine responses from total PBMCs. Unlike the murine DCs the human cells have not been phenotypically characterized in the above article. Therefore it is difficult to deduce from the current experiments whether HBD3/bacterial complexes mediate activation of human cDCS /B cells or pDCs."

Response:

We would agree with this reviewer's comments with regard to cell heterogeneity in the PBMC population as we had focussed our work on cell type characterisation using the effect of bacterial DNA in combination with hBD3 on mouse bone marrow derived cells. In the mouse we show that FLDC are stimulated by bacterial DNA and in the presence of hBD3, the level of co-stimulatory markers present on both pDC and cDC are increased. We demonstrated this effect of hBD3 on bacterial DNA (which is significantly different to the artificial CpG ligands as these synthesised oligodeoxynucelotides generally have partially or completely phosphothioated backbones to protect from nucleases and a polyG tail) and this was mediated through TLR9.

We did not however see any significant change in the negligible response of the cells to self DNA when hBD3 was present. This lack of increased response was surprising to us as others have indeed published that human self DNA does induce an increased response in pDC when combined with hBD3 (1,2). As we explain in the text, to validate the integrity of our hBD3 we then went to reproduce the result already published by Tewary et al[1] and Lande et al [2] that hBD3 could increase the response of human pDC to self DNA. The increased effect to CpG or self DNA that they see in the presence of hBD3 is reported to be mediated through human pDC and through TLR9 in human. We add a sentence (see below) to the ms to more clearly point this out. For this validation of the human work by others, we used human PBMC cells (containing a small population of pDC) as had been previously used for the human response to RNA/DNA hybrids[3]. In addition, we used CXCL10 as the read out as this has been shown to reflect the IFN response of pDC and not monocytes[4].

Based on these other works the reviewer is correct that we are assuming here that pDC are entirely responsible for the response to *bacterial* DNA that we see which is mediated through TLR9. This may not be true and we now clearly point out that although human cDC do not express TLR9 [5]other cells present do. It has been reported that TLR9 is expressed in human PBMC on NK cells (8% of PBMC); B cells (<5% of PBMC); CD4+ and CD+ T cells (<2% of PBMC) and predominantly on CD14 monocytes (45% of PBMC)[6]. pDC are part of the CD14 low population and are present in human PBMC at very small levels but do express high TLR9 and are potent producers of IFN-α.

For Peer Review

The reviewer also has a very valid point that it is possible that activation and release of IFN may trigger the other cells in the PBMC population including those not expressing TLR9. However Tewary et al [1] looked at the effect of the supernatant from human pDC (after hBD3 and fetal DNA exposure) on myeloid DC. They did indeed see activation of cDC, but this relied on incubation of 24-48 hrs and our experiments were done in a 24hr time frame. In addition, we add in the possibility that other cells in the PBMC mix might be stimulated by bacterial DNA and hBD3 to produce the CXCL10 response (see below).

New text emboldened within existing text in ms pg6-7

““HBD3 alone had no effect on cytokine expression, cell surface marker expression or viability of FLDC at the concentration used throughout this study **(5µg/ml)** (see Figure S3). However, hBD3 in combination with bacterial DNA resulted in a significant increase in the production of IFN-α and IL-6 and the expression of co-stimulatory molecules by murine FLDC compared with bacterial DNA alone (Figure 1A, B). We additionally show that hBD3 combined with bacterial DNA, significantly increased the production of CXCL10 (a cytokine described as being induced by interferon and reflecting activation of pDC[4]) by human *ex vivo* peripheral blood mononuclear cells (Figure 1C**). It is therefore likely that the bacterial DNA induced cytokine release we observe in human (as in mouse) is due to TLR9 activation of pDC in the total PBMC population. This has previously been shown with CpG in human pDC isolated from peripheral blood [2] . Human cDC do not express TLR9 [5] but other immune cells in human PBMC do express TLR9 (NK cells; B cells; CD4+ and CD8+ T cells and predominantly CD14 monocytes[6]) and these may also be activated and contribute to the increased cytokine release we observe with bacterial DNA in combination with hBD3.**

1 **Tewary, P., de la Rosa, G., Sharma, N., Rodriguez, L. G., Tarasov, S. G., Howard, O. M., Shirota, H., Steinhagen, F., Klinman, D. M., Yang, D. and Oppenheim, J. J.,**

β-Defensin 2 and 3 Promote the Uptake of Self or CpG DNA, Enhance IFN-α Production by Human Plasmacytoid Dendritic Cells, and Promote Inflammation. *J Immunol* 2013.

2 **Lande, R., Chamilos, G., Ganguly, D., Demaria, O., Frasca, L., Durr, S., Conrad, C., Schröder, J. and Gilliet, M.,** Cationic antimicrobial peptides in psoriatic skin cooperate to break innate tolerance to self-DNA. *Eur J Immunol* 2015. **45**: 203-213.

3 **Rigby, R. E., Webb, L. M., Mackenzie, K. J., Li, Y., Leitch, A., Reijns, M. A., Lundie, R. J., Revuelta, A., Davidson, D. J., Diebold, S., Modis, Y., MacDonald, A. S. and Jackson, A. P.,** RNA:DNA hybrids are a novel molecular pattern sensed by TLR9. *EMBO J* 2014. **33**: 542-558.

4 **Blackwell, S. E. and Krieg, A. M.,** CpG-A-induced monocyte IFN-gamma-inducible protein-10 production is regulated by plasmacytoid dendritic cell-derived IFN-alpha. *J Immunol* 2003. **170**: 4061-4068.

5 **Fuchsberger, M., Hochrein, H. and O'Keeffe, M.,** Activation of plasmacytoid dendritic cells. *Immunol Cell Biol* 2005. **83**: 571-577.

6 **Mortezagholi, S., Babaloo, Z., Rahimzadeh, P., Ghaedi, M., Namdari, H., Assar, S., Azimi Mohamadabadi, M. and Salehi, E.,** Evaluation of PBMC Distribution and TLR9 Expression in Patients with Systemic Lupus Erythematosus. *Iran J Allergy Asthma Immunol* 2016. **15**: 229-236.

2. The editor would also raise to the authors a requirement for the inclusion of positive controls in Figure 2 for the ability of TLR9 KO samples to react to other TLR ligands, as a control at least for cell viability. It is a basic specificity control that is required.

***Response**:*

We totally agree with this and thank the editor for pointing this out. We have included a new supplementary figure 6 where the appropriate unimpaired response of the TLR9$^{-/-}$ cells to LPS is shown and indicate this in the legend to Figure 2.

3. "Also, note that FACS profiles are not quantitative unless gates and percentages are included. The data in Fig. 3A is qualitative and not appropriate for publication."

***Response*:**

Once again we are grateful for this being pointed out and we have added the gates and percentages to figure 3A and clarified that whilst the flow cytometry plots in Fig3 A and B show representative data from individual samples for the purposes of illustration, we have provided quantification in the same part from additional independent experiments.

For Peer Review

4. **Reviewer: 1 "*The authors have demonstrated that human beta defenisn hBD3 enhances the immune response to self-DNA only in human PBMC, and not in murine cells. The murine ortholog beta defensin-14 has approx 65% sequence identity with the hDB3. So the question is will the murine BD-14 exhibit similar effects in murine cells, as seen with hBD3 in human cells? If so, this would support the functional role of these defensins in innate immune response to mammalian DNA. Either the authors can present some data with the murine BD-14 on murine cells, or if there is previous literature published discuss the effects of murine BD-14 on murine cells in the context of the data presented with hBD3 in human cells. This I believe would significantly strengthen the thesis of this manuscript.*"**

***Response**:*

We now add to the manuscript the following text to satisfy this reviewer's useful comment on page 7/8

"Defb14 is the clear orthologue of DEFB103 (hBD3) despite being only 64% identical and we have previously demonstrated that its bactericidal and chemoattractant abilities are fundamentally similar[7]. Barabas et al have shown that mouse BMDM exposed to Defb14 reveal an exacerbated response to various TLR ligands, including CpG which is known to activate TLR9. This implies that the increased response to bacterial DNA by these defensin peptides is conserved between human and mouse (8)."

7. **Taylor, K., Clarke, D. J., McCullough, B., Chin, W., Seo, E., Yang, D., Oppenheim, J., Uhrin, D., Govan, J. R., Campopiano, D. J., Macmillan, D., Barran, P. E. and Dorin, J. R.,** Analysis and separation of residues important for the chemoattractant and antimicrobial activities of beta -defensin 3. *J.Biol.Chem.* 2008. **283:**: 6631.-6639.
8. **Barabas, N., Röhrl, J., Holler, E. and Hehlgans, T.,** Beta-defensins activate macrophages and synergize in pro-inflammatory cytokine expression induced by TLR ligands. *Immunobiology* 2013. **218**: 1005-1011.

5. **Referee 1: Minor comments:** "On Page 1, in the introduction, references to support the sentence "HDP are cationic peptides with amphipathic structures enabling them to rapidly enter cells" need to include other HDPs and not just hBD3"

Response:

For Peer Review

Thank you, we have done this and added reference to another cationic, amphipathic HDP cell penetrating peptide LL-37 (Lau et al 2005).

6. **“Please provide a rationale for the concentrations used for the stimulants, i.e. 1 ug/ml for genomic DNA and 5 ug/ml for hBD3, in this manuscript.”**

Response:

We used 1 µg/ml of bacterial DNA as this has been used routinely by others to precipitate a response in pDC and was in keeping with levels derived from a bacterial infection. We used 5 µg/ml for the peptide as this had been shown by to be an appropriate amount in FLDC to increase the response to the E.coli DNA and not be in the range that might be damaging to the cells (Figure S3) and was still at a physiological relevant level under infection conditions. We now include in Supplementary figure 4 the concentration titration of hBD3 against 1 µg/ml E. coli DNA.

7. **In Supplementary Figure 3: replace ‘at the concentration used throughout this study’ with the concentration in parenthesis (5 ug/ml).**

Response:

Yes, thank you we have changed this now.

8. **Reviewer: 2 Minor comments**. “Cell surface marker and gating strategies and for pDC and cDCs are inadequate. Conventional DCs are generally B220 negative rather than B220 low.”

Response:

Sorry, we have corrected this (we had B220 negative in the figure, not sure how it became “low” in text).

9. **Supplementary figure 1. Expression of costimulatory molecules is not clear as to which population is being analyzed (cDCs or pDCs)**

For Peer Review

Response

We apologise and have now clarified this in S1

10. "HBD3 mediated complex/aggregate formation of CpG DNA (similar to bacterial DNA) has already been documented, hence the data is a confirmation of previously published work."

Response

Please refer to point 1 for further information on this. Previously others indicated that human self DNA and also CpG DNA when combined with hBD3 produced an increased TLR9 mediated response in human cells. CpG is different to bacterial DNA and we explain that in point 1 and in the text. Here we show that bacterial DNA is detected by mouse pDC and this activation is increased in the presence of hBD3 and this is entirely mediated by TLR9. We also show a similar increase in detection of bacterial DNA in the presence of hBD3 in human cells. hBD3 facilitates entry of both self and bacterial DNA into the cells but we do not see an increase in detection of self DNA in mouse cells when they are exposed to hBD3. We can confirm that in human cells we see what has already been reported. Where we refer to already reported work we make this clear with the reference.

Prof. Julia Dorin,
MRC Centre for Inflammation Research,
University of Edinburgh
Queen's Medical Research Institute,
47 Little France Crescent, Edinburgh EH16 4TJ,
Scotland, UK

Tel: +44 (0)131 242 6590

Second Editorial Decision

10-Jan-2017

Dear Dr. Dorin,

For Peer Review

It is a pleasure to provisionally accept your manuscript entitled "Human Î²-defensin 3 increases the TLR9-dependent response of plasmacytoid dendritic cells to bacterial DNA." for publication in the European Journal of Immunology. For final acceptance, please follow the instructions below and return the requested items as soon as possible as we cannot process your manuscript further until all items listed below are dealt with.

Please note that EJI articles are now published online a few days after final acceptance (see Accepted Articles: http://onlinelibrary.wiley.com/journal/10.1002/(ISSN)1521-4141/accepted). The files used for the Accepted Articles are the final files and information supplied by you in Manuscript Central. You should therefore check that all the information (including author names) is correct as changes will NOT be permitted until the proofs stage.

We look forward to hearing from you and thank you for submitting your manuscript to the European Journal of Immunology.

Yours sincerely,
Nadja Bakocevic

on behalf of
Prof. Kenneth Murphy

Dr. Nadja Bakocevic
Editorial Office
European Journal of Immunology
e-mail: ejied@wiley.com
www.eji-journal.eu

For Peer Review
